# Internet-Based Lifestyle Intervention to Prevent Type 2 Diabetes Through Healthy Habits: Design and 6-Month Usage Results of Randomized Controlled Trial

**DOI:** 10.2196/15219

**Published:** 2020-08-11

**Authors:** Marja Harjumaa, Pilvikki Absetz, Miikka Ermes, Elina Mattila, Reija Männikkö, Tanja Tilles-Tirkkonen, Niina Lintu, Ursula Schwab, Adil Umer, Juha Leppänen, Jussi Pihlajamäki

**Affiliations:** 1 VTT Technical Research Centre of Finland Ltd Espoo Finland; 2 Institute of Public Health and Clinical Nutrition School of Medicine University of Eastern Finland Kuopio Finland; 3 Endocrinology and Clinical Nutrition Department of Medicine Kuopio University Hospital Kuopio Finland

**Keywords:** digital behavior change intervention, internet intervention, web-based intervention, behavior change support system, type 2 diabetes, habit

## Abstract

**Background:**

Type 2 diabetes can be prevented through lifestyle changes, but sustainable and scalable lifestyle interventions are still lacking. Habit-based approaches offer an opportunity to induce long-term behavior changes.

**Objective:**

The purposes of this study were to describe an internet-based lifestyle intervention for people at risk for type 2 diabetes targeted to support formation of healthy habits and explore its user engagement during the first 6 months of a randomized controlled trial (RCT).

**Methods:**

The app provides an online store that offers more than 400 simple and contextualized habit-forming behavioral suggestions triggered by daily life activities. Users can browse, inspect, and select them; report their performances; and reflect on their own activities. Users can also get reminders, information on other users’ activities, and information on the prevention of type 2 diabetes. An unblended parallel RCT was carried out to evaluate the effectiveness of the app in comparison with routine care. User engagement is reported for the first 6 months of the trial based on the use log data of the participants, who were 18- to 70-year-old community-dwelling adults at an increased risk of type 2 diabetes.

**Results:**

Of 3271 participants recruited online, 2909 were eligible to participate in the RCT. Participants were randomized using a computerized randomization system to the control group (n=971), internet-based intervention (digital, n=967), and internet-based intervention with face-to-face group coaching (F2F+digital, n=971). Mean age of control group participants was 55.0 years, digital group 55.2 years, and F2F+digital 55.2 years. The majority of participants were female, 81.1% (787/971) in the control group, 78.3% (757/967) in the digital group, and 80.7% (784/971) in the F2F+digital group. Of the participants allocated to the digital and F2F+digital groups, 99.53% (1929/1938) logged in to the app at least once, 98.55% (1901/1938) selected at least one habit, and 95.13% (1835/1938) reported at least one habit performance. The app was mostly used on a weekly basis. During the first 6 months, the number of active users on a weekly level varied from 93.05% (1795/1929) on week 1 to 51.79% (999/1929) on week 26. The daily use activity was not as high. The digital and F2F+digital groups used the app on a median of 23.0 and 24.5 days and for 79.4 and 85.1 minutes total duration, respectively. A total of 1,089,555 habit performances were reported during the first 6 months. There were no significant differences in the use metrics between the groups with regard to cumulative use metrics.

**Conclusions:**

Results demonstrate that internet-based lifestyle interventions can be delivered to large groups including community-dwelling middle-aged and older adults, many with limited experience in digital app use, without additional user training. This intermediate analysis of use behavior showed relatively good engagement, with the percentage of active weekly users remaining over 50% at 6 months. However, we do not yet know if the weekly engagement was enough to change the lifestyles of the participants.

**Trial Registration:**

ClinicalTrials.gov NCT03156478; https://clinicaltrials.gov/ct2/show/NCT03156478

## Introduction

### Background

The prevalence of diabetes is rapidly increasing globally and is now almost 10% among adults aged 25 years and older [1], with type 2 accounting over 90% of the cases [2]. According to the International Diabetes Federation, the cause of type 2 diabetes is not completely understood, but it is largely connected to excess body weight, increasing age, ethnicity, and family history [3]. Type 2 diabetes can be prevented or delayed by influencing modifiable risk factors through healthy lifestyles [3].

The key challenges in type 2 diabetes prevention are scaling up interventions, selecting the most appropriate intervention, tailoring interventions to different populations and settings, and ensuring clinically meaningful, cost-effective outcomes [4]. There are increasing efforts to provide readily accessible, cost-effective type 2 diabetes interventions to the general public [5]. Interventions using digital technology are of special interest because they may be easier to disseminate and maintain compared with diabetes prevention programs delivered by health care professionals or peers [5].

Systematic reviews have shown that digital interventions can be effective, and effectiveness is mediated by factors related to health behavior change and intervention characteristics related to user engagement in the intervention [6]. Development of digital behavior change interventions should be driven by direct and indirect evidence and behavior change theory [7]. While many different theories, approaches, and techniques have been used in behavior change research, most digital behavior change interventions fail to take habitual behavior into account [8]. Habits are central in changing health behaviors because an estimated 50% to 95% of daily life behaviors are habits, performed relatively automatically with little thought or regard to current goals or intentions [9,10].

Habit-formation approaches promote the repetition of behavior until it becomes habitual, provide context cues to trigger the behavior, and give rewards that help strengthen the association between the context cues and the behavior [11]. Promoting the repetition of behaviors is about creating opportunities for and encouraging frequent repetition of specific responses (eg, through visual advertisements of providing possibilities to rehearse the new habit) [11]. According to Wood and Neal [11], the provided context cues should be stable and can include times of day, locations, prior actions in a sequence, or presence of other people. People can be encouraged to create plans (ie, implementation intentions) to perform a behavior in a given context. Interventions can also tie a new healthy behavior to an existing habit, which is called piggybacking. Provision of rewards may help in habit forming especially at the early stages of habit formation [11]. A recent review on digital behavior change interventions shows that only 3 interventions out of 85 targeted formation of new healthy habits [12].

Another important factor for sustained engagement in behavior change is the quality of motivation [13]. Self-determination theory (SDT) [14] defines a continuum from controlled to autonomous motivation, where controlled motivation is driven by external factors such as sanctions, rewards, social pressure, etc, and autonomous motivation by internal factors such as individual values and enjoyment, thereby fulfilling the individual’s basic psychological needs: perceptions of autonomy, control or self-efficacy, and relatedness [14]. Interventions to prevent type 2 diabetes are based on evidence from a limited set of lifestyle objectives describing what [15,16] people should achieve, but programs could provide individuals freedom of choice on how to reach these objectives. If individuals could select in which order to start and from whom to receive the necessary support, it would increase their autonomy in the selection of the changes they pursue, their sense of self-efficacy resulting from achievement, and their feelings of relatedness with peers or significant others, resulting in improved fit with their daily lives and higher odds for maintenance.

Using habit-based approaches and SDT as the behavior change theories to guide digital intervention development holds great promise to induce long-term behavior changes and bring lasting public health benefits.

### Objectives

The purpose of this study was to describe an internet-based intervention targeted for people at risk for type 2 diabetes to support formation of healthy habits and explore use behavior during the first 6 months of a randomized controlled trial (RCT).

## Methods

### Design and Randomization

The internet-based intervention, called the BitHabit app, was developed in a national research project studying the real-world implementation of evidence-based type 2 diabetes prevention programs. The study was a 1-year unblinded parallel RCT [NCT03156478] conducted across 3 regions in Finland (Northern Savo, Päijät-Häme, and Southern Carelia). The detailed protocol and design for the study were reported elsewhere [17].

Participants in the trial were randomized using a computerized randomization system, and they were allocated to one of 3 groups: (1) control group, (2) internet-based intervention (digital), or (3) internet-based intervention with face-to-face group coaching (F2F+digital). Allocation to the intervention groups was made 1:1:1 using a computerized randomization system. This is an intermediate analysis focusing on the use behavior of the participants allocated to the intervention groups.

Participants were recruited online between March 2017 and February 2018 through a digital risk-screening tool that was provided through the project’s website. Participants were attracted to the website by varied means including social media, newspapers, radio, television, websites, health care and social service units, and community pharmacies in collaboration with municipal services, employers, patients associations, and other nongovernmental organizations [17]. Individuals identified to be eligible and willing to participate in the study were given instructions on how to contact a nurse in a local health care center for examination visits.

The study was approved by Research Ethics Committee of the Hospital District of Northern Savo (statement number: 467/2016). Written informed consent to participate in the study and for the use of data from national health care registers was obtained from all participants. The informed consent procedure is described in detail in the trial protocol article [17]. The study is conducted according to the Responsible Conduct of Research by the Finnish Advisory Board on Research Integrity and the Declaration of Helsinki.

### Participants

The inclusion criteria were (1) aged between 18 and 70 years; (2) increased risk of type 2 diabetes based on a Finnish Diabetes Risk Score ≥12 points [18] or a history of gestational diabetes or repeated impaired fasting glucose (fasting plasma glucose 6.1 to 6.9 mmol/L) or impaired glucose tolerance (2-hour plasma glucose 7.8 to 11.0 mmol/L in 2-hour oral glucose tolerance test); (3) living in the province of Northern Savo, Päijät-Häme, or Southern Carelia; (4) access to a computer, smartphone, or tablet with internet connection; (5) having a phone number of their own; and (6) having adequate Finnish language skills. The exclusion criteria were (1) type 1 or type 2 diabetes; (2) pregnancy or breastfeeding; and (3) active cancer or less than 6 months from cancer treatment.

### Requirements for the Internet-Based Intervention

The overall development of the internet-based intervention was guided by the Medical Research Council Guidelines on Development of Complex Health Interventions [19]. Identifying the evidence base from the literature proceeded in parallel with ideation, benchmarking, and prototyping. After feasibility testing, changes were made in both content and functionality.

The two lines of behavior change theory that formed the basis of app development were habit formation approaches and SDT. These approaches were considered to be suitable to support maintenance of behavior change, which is a challenge in lifestyle interventions [13]. SDT and especially autonomy support have been associated with higher effectiveness in the long-term, and habit-based approaches also show promise in this respect [13].

In the habit formation approach, it is important to offer tiny behaviors that can be easily repeated and expanded from one-time or occasional behaviors to repeated sequences of behaviors and finally to permanent behaviors [20]. Furthermore, the frequent repetition of these behaviors needs to be sufficiently supported in a stable context in order for the users to be able to form a cognitive association between context cues and responses and provide some kind of reward to strengthen the association [11]. Thus, the app was designed to promote selection of tiny behaviors that were linked to a specific trigger and boost execution of the behaviors until they become automatic habits.

SDT as the evidence base also provided some key requirements for the app. In order to promote autonomy, a broad selection of behaviors was required to foster freedom of choice. To enhance self-efficacy, the behaviors needed to be feasible for the users, and they also had to be behaviors that users were already familiar with. Finally, enhancing relatedness and sense of community was a challenge. Traditionally, it has been perceived as the sense of being respected, understood, and cared for by health care professionals, forming experiences of connection and trust [21]. We decided to enhance relatedness and sense of community through other participants of the study by providing a possibility for the users to learn about other users’ activities in the app.

Other requirements were derived from benchmarking, feasibility testing with a group of end users representing people at risk for type 2 diabetes, and the research consortium that had real-time access to the app during its development. There was a need to design a scalable app that could be automatically taken into use after randomization by community-dwelling middle-aged and older adults—many with limited experiences in digital app use—without any additional support. It was found that smartphone use is much less common among people aged over 45 years, especially among women [22]. Thus, it was necessary to implement a web-based app suitable for all smart devices without requiring installment of a native app. The feasibility study showed that participants did not always know how to use their smartphones and had difficulties with wireless networks, passwords, and touch screens. In addition, they were used to using search engines for accessing websites and not the address bar. Thus, there was a need to develop an easy way to access the app.

### Internet-Based Intervention

We decided to provide an experience similar to online shopping, which most adults are familiar with [22]. Rather than traditional health apps, we took the online store as our model, with the idea of offering health behaviors as the products organized in different departments or categories. The BitHabit app provides an extensive habit library we called a store of habits that was developed by translating lifestyle guidelines and recommendations into simple habit-forming behavioral suggestions, which we named BitHabits.

**Figure 1:**
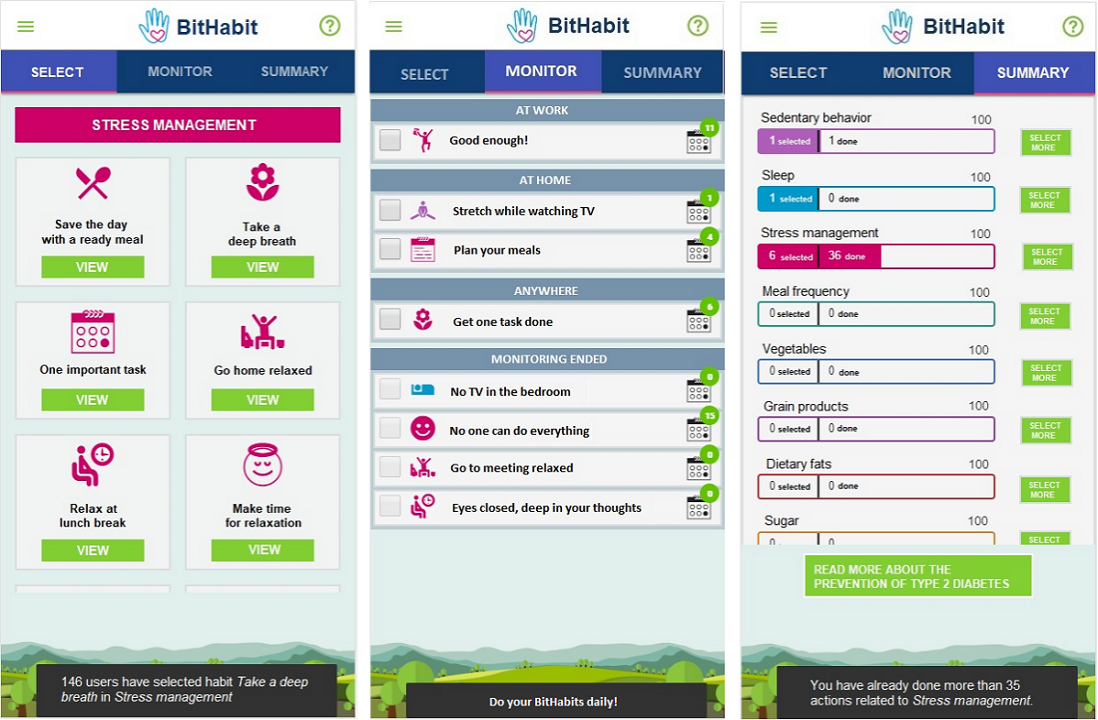
Three main views of the BitHabit app.

Users could log in to the app via a personalized link that they received via email and text message. When they clicked the link, the app opened up in a web browser. When users logged in for the first time, a brief health behavior questionnaire was launched. After that, they entered the app, which has 3 main views: (1) select view for browsing, inspecting, and selecting habits; (2) monitor view for reporting performances; and (3) summary view for reflecting on activities (Figure 1).

At their first visit, users landed in the select view where they could browse, inspect, and select BitHabits. Each BitHabit has a brief title, a more detailed description, and a health fact derived from the existing knowledge. Selected BitHabits appeared in the monitor view, a shopping basket. To promote execution and automation, BitHabits were presented per physical contexts common in the users’ everyday lives; home, work, grocery store, and so on.

**Figure 2:**
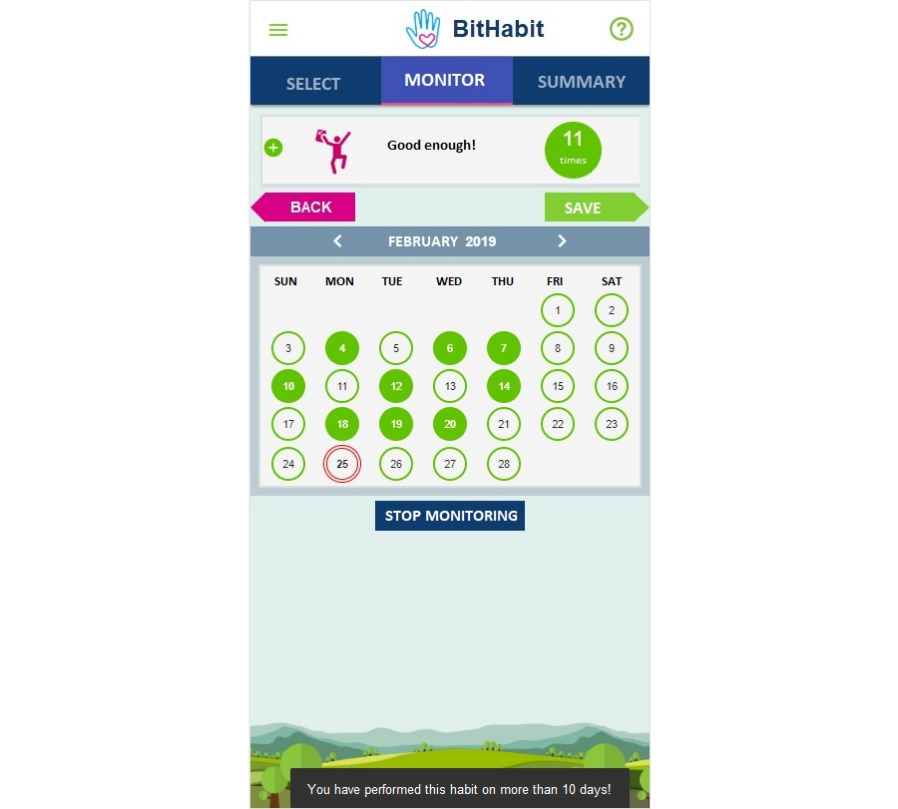
Calendar-functionality enables reporting performances.

After users made at least one selection, they landed in the monitor view. Users were expected to report the performance of BitHabits on a daily basis, but they could also add performances afterward through the calendar view (Figure 2). Intended dose was not recommended for them. An additional feature included in the calendar view was a choice to stop monitoring a BitHabit.

The summary view presented an overview of user selections and performances per lifestyle category in a horizontal bar graph. The left side of the bar showed the number of selections per category. As soon as users made at least one selection, the bar color changes and seems full. The right side of the bar showed the number of performances. The maximum number for performances was 100, but users could collect more if they liked. Users were able to go directly from this view to browsing, inspecting, and selecting new BitHabits.

Use instructions with privacy notice were available through the green question mark icon. In addition, pop-up functionality sent use instructions during the first use sessions and provided simple feedback. Feedback consisted of anonymous information on other users’ selections during the habit selection (eg, 160 users have selected this BitHabit), and automatic feedback related to a certain habit (eg, you have been performing this BitHabit for 30 days) or number of performances in different lifestyle categories (eg, you have already performed over 35 BitHabits from the meal frequency category).

Reminders were sent when (1) user received a link to the intervention app but was not logged in, (2) user was logged in but had not made any selections, (3) user made selections but had not reported them, and (4) user had logged in at least once but had not used the app for a week. Reminders 2 and 3 were added shortly after the app was launched.

The order of the categories in the user interface was determined by the brief health behavior questionnaire presented in the beginning. The categories where the improvement potential was highest were presented first. A system-level description of the BitHabit app is available in Multimedia Appendix 1.

**Figure 3:**
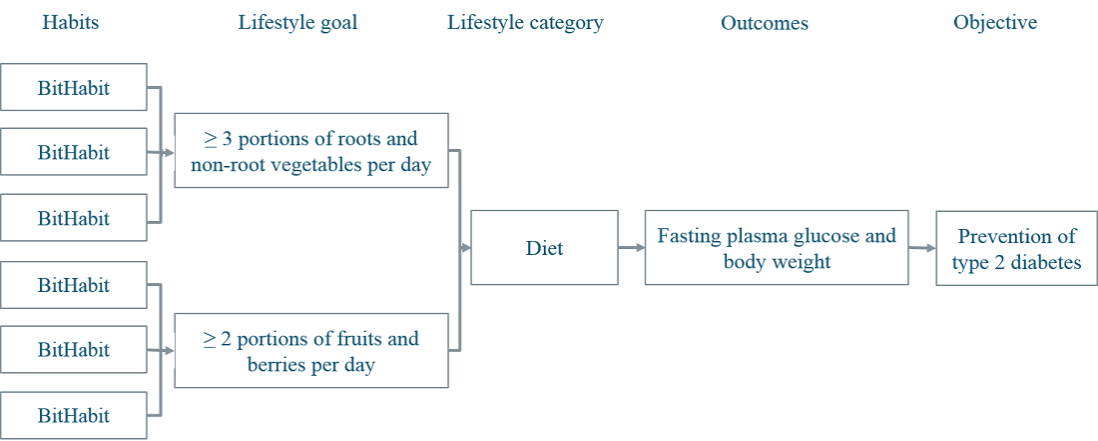
Simplified content-related logic model.

The store contained 489 BitHabits divided into 13 categories: meal frequency (43 habits), vegetables (53 habits), dietary fat (38 habits), grain products (24 habits), sugar (20 habits), alcohol and other drinks (19 habits), everyday physical activity (64 habits), conditioning physical activity (68 habits), sedentary behavior (36 habits), sleep (42 habits), positive mood (37 habits), stress management (23 habits), and nonsmoking (22 habits). Sleep, stress management, and positive mood were incorporated into the design with the more traditional type 2 diabetes risk factors because of increasing evidence of their relevance to cardiometabolic diseases and increasing prevalence of comorbidity between type 2 diabetes and common mental disorders [23,24]. We also wanted to promote the use of the app among those who were not comfortable in making changes related to diet or physical activity. The sample of a content-related logic model is presented in Figure 3.

### Internet-Based Intervention With Face-to-Face Group Coaching

Both groups got access to the BitHabit app in the same way, through the personalized link they received via email and text message. In addition, the participants allocated to the face-to-face coaching plus digital intervention (F2F+digital) group were invited to participate in group coaching consisting of 6 meetings organized in local health care centers. Topics of these meetings were type 2 diabetes, rhythm of daily life, healthy diet, physical activity, automating activity to everyday life, and self-evaluation of program outcomes [17]. Internet-based intervention and group coaching share the same overarching behavior change theory, SDT [14], and they share the same lifestyle goals [17].

The groups were facilitated by nurses or other health care professionals. The BitHabit app was introduced to them during their training program, and they also got access to the app. Later they had an opportunity to participate in a professional development day where app use as part of the group coaching was discussed. However, group facilitators were not expected to give advice related to app use. In the participant workbook, app use was mentioned very briefly using app-related tasks such as searching a habit related to the topic at hand.

### Measurements and Data Analysis

For this intermediate analysis, use data from the BitHabit app during the first 6 months of the study were available. The BitHabit app automatically collected log files of user interactions, selected habits, and habit performances in the app. The user interactions log contained a time stamped log of each page view in the app. The log of selected habits contained all habit selections during the course of the app use. The habit performance log collected all habit performances as marked by the user in the monitor and calendar views along with dates when the user claims to have performed the habit. In addition, a limited set of baseline data was available to describe the demographics of the intervention groups. The other measurements of the study are described elsewhere [17].

Relevant variables included use sessions, use days, duration of use, percentage of users accessing the app on a daily and weekly level, number of visits to each view of the app, start times of the identified use sessions, and selected and performed habits per categories. The research questions for engagement in this intermediate analysis are focused on describing the overall use activity and use behavior:

- How well were participants able to access the app and try out its basic functionalities?
- How actively was the app used over the course of the first 6 months?
- In which ways was the app used and how did the use evolve during the first 6 months?
- How were the app use times distributed during the day?
- How were the selections and performances of habits distributed among the different categories?
- Were there any differences between the intervention groups?

The analysis covers the first 6 months, or more accurately 26 weeks, of the intervention, starting from the date the participants received the invitation to the app. As the distributions of the use metrics are very skewed, medians and interquartile ranges (IQR) of the use metrics are reported. Comparison of the use metrics between groups was done with Mann-Whitney *U* tests. The analyses were conducted with Matlab R2017a (The Mathworks Inc) and SPSS Statistics version 26 (IBM Corp). Statistical significance was set at *P*<.05.

## Results

### Participant Recruitment

**Figure 4:**
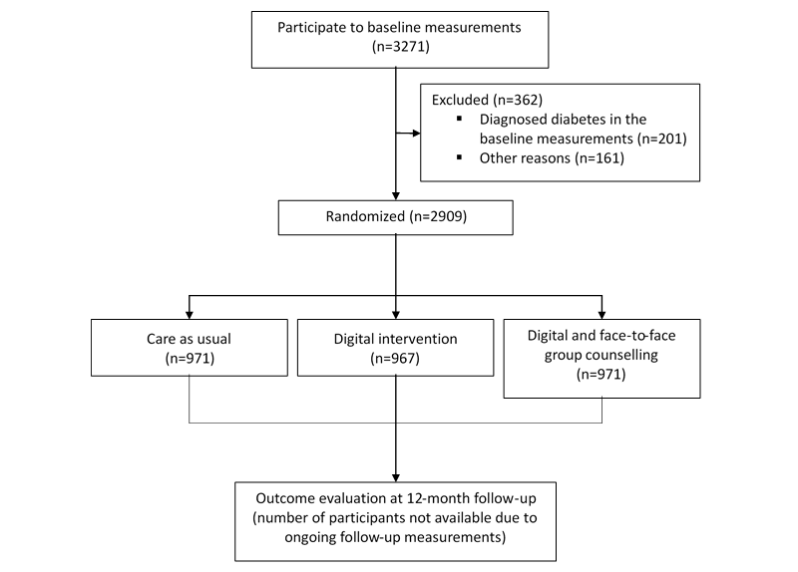
Flow diagram of the Stop Diabetes intervention study [17].

A total of 3271 individuals were recruited to participate in the study. Of these, 362 participants were excluded, 201 due to being diagnosed with type 2 diabetes in the baseline measurements and 161 for other reasons. Finally, 2909 participants were randomized, of which 971 were allocated to the control group, 967 to the internet-based intervention, and 971 to the internet-based intervention with face-to-face group coaching. The flow diagram of the Stop Diabetes intervention study is presented in Figure 4.

### Participant Characteristics

Baseline participant characteristics are reported in Table 1. The mean age of the control group was 55.0 (SD 9.9) years, digital group 55.2 (SD 9.9) years, and F2F+digital 55.2 (SD 10.1) years; 81.1% (787/971) of the control group, 78.3% (757/967) of the digital group, and 80.7% (784/971) of the F2F+digital group participants were women. More than half of the participants were working (1693/2909, 58.20%), and almost one-third (803/2909, 27.60%) were retired.

**Table 1:** Participant characteristics (n=2909).

Characteristics		Control group (n=971)	Digital intervention (n=967)	Face-to-face coaching and digital intervention (n=971)
Age in years, mean (SD)		55.0 (9.9)	55.2 (9.9)	55.2 (10.1)
**Gender, n (%)**				
	Women	787 (81.1)	757 (78.3)	784 (80.7)
	Men	184 (18.9)	210 (21.7)	187 (19.3)
Weight (kg), mean (SD)		87.1 (16.9)	86.5 (17.4)	85.7 (17.0)
Body mass index (kg/m^2^), mean (SD)		31.3 (5.4)	31.0 (5.4)	30.9 (5.4)
Waist circumference (cm), mean (SD)		102 (13.2)	101.5 (13.3)	101.1 (13.0)
**Education, n (%)**				
	Elementary school	45 (4.6)	45 (4.7)	50 (5.2)
	Middle school	24 (2.5)	18 (1.9)	35 (3.6)
	Vocational school	223 (23.0)	239 (24.7)	214 (22.0)
	High school	44 (4.5)	37 (3.8)	39 (4.0)
	Institute degree	287 (29.6)	270 (27.9)	270 (27.8)
	Bachelor’s degree	195 (20.1)	195 (20.2)	221 (22.8)
	Higher academic degree	153 (15.8)	163 (16.9)	142 (14.6)
**Work status, n (%)**				
	Wage earner	522 (53.8)	504 (52.1)	489 (50.4)
	Entrepreneur	55 (5.7)	60 (6.2)	63 (6.5)
	Unemployed	56 (5.8)	50 (5.2)	54 (5.6)
	Student	21 (2.2)	25 (2.6)	23 (2.4)
	Pensioner	254 (26.2)	275 (28.4)	274 (28.2)
	Other	63 (6.2)	53 (5.5)	68 (7.0)
**Marital status, n (%)**				
	Married	616 (63.4)	608 (62.9)	582 (59.9)
	Cohabitation	106 (10.9)	120 (12.4)	124 (12.8)
	Registered relationship	1 (0.1)	0 (0)	1 (0.1)
	Unmarried	64 (6.6)	76 (7.9)	83 (8.6)
	Divorced	154 (15.9)	140 (14.5)	143 (14.7)
	Widowed	30 (3.1)	23 (2.4)	38 (3.9)

### Access to BitHabit App and Trying Out the Basic Functionality

Almost all participants were able to access the app with their own smart device and try out the basic functionality of selecting habits and reporting them. Of the participants allocated to the digital and F2F+digital groups, 99.53% (1929/1938) logged in to the app at least once; they will be henceforth called app users. Of the app users, 98.55% (1901/1929) selected at least one habit. At least one habit performance was reported by 95.13% (1835/1929) of app users.

### Use Activity During the First Six Months

**Figure 5:**
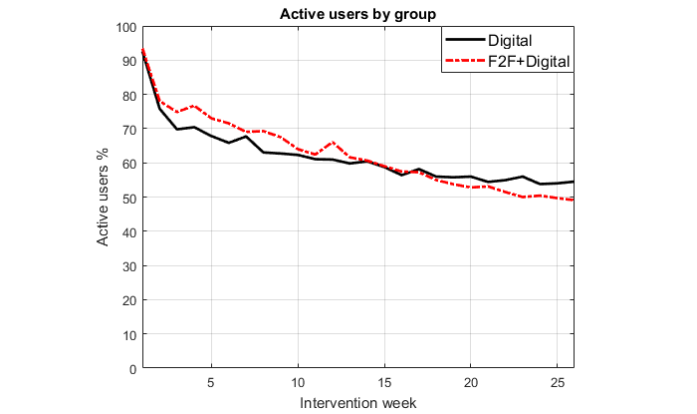
Percentage of active users per intervention week.

During the first 6 months, the number of active users on a weekly level varied from 93.05% (1795/1929) in week 1 to 51.79% (999/1929) in week 26. The daily use activity was not as high; on any given day during the first 6 months, a median of 17.21% (332/1929; IQR 15.3%-20.3%) of users accessed the app. Figure 5 presents the percentage of active users by group per intervention week.

Cumulative use metrics for the two groups are summarized in Table 2. The digital and F2F+digital groups used the app on a median of 23.0 and 24.5 days and for 79.4 and 85.1 minutes total duration, respectively.

**Table 2:** Cumulative use metrics for the first 6 months.

Use metric	Digital, median (IQR)	F2F+Digital^a^, median (IQR)
Use sessions	26.0 (13.0-48.0)	28.0 (13.8-50.0)
Use days	23.0 (12.0-42.0)	24.5 (12.0-42.0)
Use weeks	18.0 (8.00-23.0)	18.0 (10.0-23.0)
Session duration, average (minutes)	3.05 (1.79-5.14)	3.25 (1.95-5.12)
Total duration (minutes)	79.4 (37.0-167.0)	85.1 (37.3-188.4)
Selected habits	24.0 (13.0-44.0)	24.0 (12.0-45.0)
Reported performances	263.0 (59.8-703.3)	277.0 (66.0-747.5)
Days with performances	45.0 (14.0-131.0)	52.0 (17.0-131.0)
Categories of reported habits	9.0 (6.0-12.0)	9.0 (5.0-12.0)

^a^F2F+digital: face-to-face coaching plus digital intervention.

### Ways of Use

Most page views were related to the use of the monitor view (ie, monitoring and reporting of performed habits). Only during the first month was the use of the select view to browse, inspect, and select habits more popular. The summary view was not used very frequently compared with the other views.

The use of the monitor view increased over time as more use focused on reporting performances and also because the view became the landing page as soon selections were made. The use of the calendar view under the monitor view increased over time, probably indicating that users started marking more performances through the calendar view and thus, marking several days’ performances at a time instead of marking them daily. The use of the select view (ie, browsing, inspecting and selecting new habits) decreased over time. Table 3 shows the distribution of views visited over the 6 months.

**Table 3:** Distribution of views visited and changes over months.

View	Month 1	Month 2	Month 3	Month 4	Month 5	Month 6
Browse	19.97	11.77	9.23	7.89	7.52	6.80
Browse/Inspect	30.85	15.74	11.99	10.22	9.34	8.06
Monitor	34.47	47.15	51.72	54.19	55.92	57.25
Monitor/Calendar	9.74	19.22	21.15	21.62	21.60	22.44
Reflect	4.97	6.12	5.91	6.08	5.61	5.46

### App Use Times During the Day

The use of the BitHabit app was quite well spread over the assumed waking hours of the users. The most active hours were from 8:00 pm to 10:00 pm, when 18.5% of the sessions were started. Table 4 presents a summary of use with respect to the time of day in the intervention groups.

**Table 4:** Use of the BitHabit app according to the time of day.

Time of day	Percentage of sessions
00:00-00:59	0.84
01:00-01:59	0.33
02:00-02:59	0.14
03:00-03:59	0.16
04:00-04:59	0.29
05:00-05:59	0.66
06:00-06:59	1.84
07:00-07:59	2.88
08:00-08:59	4.34
09:00-09:59	4.37
10:00-10:59	5.39
11:00-11:59	5.61
12:00-12:59	5.17
13:00-13:59	4.67
14:00-14:59	4.54
15:00-15:59	4.82
16:00-16:59	5.22
17:00-17:59	5.39
18:00-18:59	6.14
19:00-19:59	7.33
20:00-20:59	9.04
21:00-21:59	9.48
22:00-22:59	7.53
23:00-23:59	3.84

### Selections and Performances in Different Habit Categories

Most habits were selected from the stress management, positive mood, and vegetables categories. In addition, meal frequency, everyday physical activity, and alcohol and other drinks were selected by over 700 users in both groups. A total of 1,089,555 habit performances were reported during the first 6 months of the study. Table 5 presents a detailed summary of habit selections and performances. For each habit category, the number of selections and number of users who selected habits from the category, number of habit performances, and number of users who performed habits from each category are presented for both groups.

### Differences Between the Intervention Groups

There were no significant differences in use metrics between the groups with regard to cumulative use metrics (Table 2), and selected habit categories were very similar in both groups. In the digital group, most performances were reported in the meal frequency, positive mood, and stress management categories. In the F2F+digital group, most performances were reported in the vegetables, stress management, and meal frequency categories (Table 5). In both groups, the most popular categories achieved over 60,000 reported habit performances.

**Table 5:** The number of selections and users and the number of habit performances and users per group.

Habit category	Selections, digital		Selections, F2F+digital^a^		Performances, digital		Performances, F2F+digital	
	Total	Users	Total	Users	Total	Users	Total	Users
Meal frequency	2652	708	2558	709	64,244	656	60,248	643
Vegetables	3148	732	3125	739	57,509	672	65,319	681
Grain products	1843	622	1825	631	37,497	559	36,677	564
Dietary fat	2809	565	2721	670	46,583	596	46,628	617
Sugar	1938	614	1905	607	16,491	532	17,113	528
Conditioning physical activity	2653	688	2575	695	29,930	610	30,954	631
Everyday physical activity	2854	708	2796	696	29,197	625	32,528	615
Sedentary behavior	1931	620	1951	629	19,625	529	21,524	534
Alcohol and other drinks	2460	722	2375	737	56,815	678	52,200	689
Nonsmoking	417	211	286	176	5781	172	4155	142
Sleep	2400	647	2327	641	59,830	584	54,313	584
Stress management	4402	807	4340	815	61,664	737	63,890	755
Positive mood	3407	718	3254	688	63,212	646	55,628	620

^a^F2F+digital: face-to-face coaching plus digital intervention.

## Discussion

### Achievement of Objectives

The purpose of this study was to describe an internet-based lifestyle intervention targeted to support healthy habits and to explore use behavior during the first 6 months. We developed the idea of an app providing an online store offering habit-forming behavioral suggestions that could be easily adopted in everyday life. We were able to recruit over 3000 participants, of whom 1938 were allocated in the active intervention groups using the BitHabit app. Participants were mainly middle-aged and older adults, and the majority of them were women. They were a relatively typical participant group for a type 2 diabetes prevention program [25]. Our research questions for engagement in this intermediate analysis were focused on describing the overall use activity and use behavior.

### Principal Findings

Among our participants, almost all (1929/1938) opened the app at least once, and almost all who logged in selected at least one habit and reported at least one performance. Based on this, it can be concluded that the app was accessible by our target group.

On a weekly level, the percentage of active users varied from 93.1% to 51.8%. This use activity compares favorably with previous studies of similar technologies. For example, the shape of the graph of active users resembles the one for mobile apps in Mattila et al [26], but their percentage of active users was only about 30% at 6 months. The difference in favor of the BitHabit app may partly be explained by the reminder feature. In Kaipainen et al [27], where a publicly available online healthy eating and weight loss program was studied, 25% of the participants who started the program returned for a follow-up. In Helander et al [28], where a free mobile app for dietary self-monitoring was studied, 2.58% used the app actively. On a daily level, use activity of the BitHabit app was lower than expected, with a median of 17.2% of users accessing the app on any given day during the first 6 months. This implies that we failed to create a daily pattern of use of the app but supported weekly use instead. However, we cannot yet say whether daily use of the app is actually required. The relationship between use and health-related outcomes may be complex, and sufficient engagement with the intervention to achieve intended outcomes (ie, effective engagement) needs to be determined empirically, keeping in mind that it can also be dependent on individual users’ characteristics and context of use [29,30]. Decreasing use may not always imply disengagement from the intervention; it may be due to achievement of desired health outcomes or behavior changes [31]. An early study on mobile self-monitoring found that after a period of frequent self-monitoring, participants felt they learned to self-monitor without the app, which decreased the frequency of monitoring, especially for food-related events [32].

The analysis of ways of use showed that the app was mostly used for monitoring and reporting the performed BitHabits. Only during the first month were browsing, inspecting, and selecting habits more popular than monitoring and reporting. This was expected because the monitor view was the landing page (ie, first page that opened for the users every time they entered the app), and users were supposed to report their habits on a daily or weekly basis. The least visited view was the view presenting a summary of the all performances in different categories. Our original objective was to provide feedback of performances in a simple visual way with elements of gamification—such as a colored bar that could be filled by performances—that would also serve as a booster for selecting and reporting more BitHabits. However, our findings suggest that the view with its functions was not able to fulfill the objectives and hence requires further development.

The analysis of use times showed that use of the BitHabit app was quite well spread over the assumed waking hours of the users, and the most active hours were in the evening. Context recognition and timely reminders would encourage use throughout the day, but we were not able to provide such features because wide accessibility through a web-based app was deemed more important. Also, the evidence related to reminders is not indisputable. Based on the literature, time cues might even prevent habit formation. According to Lally et al [33], prospective memory research indicates that situations permit external cueing of an intended action whereas time cues require monitoring to identify the time to act [34].

The most actively selected habit categories were stress management, positive mood, and vegetables and fruits in both groups, and it was a bit surprising that stress management and positive mood were among the top 3 categories. Originally these not-so-obvious type 2 diabetes risk factors were incorporated into the design because there was increasing evidence of their relevance to cardiometabolic diseases, and we also wanted to promote the use of the app among those who are not comfortable in making changes related to diet or physical activity. On the other hand, the prevalence of stress and other mental health issues is rapidly increasing. Mental and behavioral disorders was the largest disease group causing disability leading to disability pension in Finland in 2018, causing 43% of all disability pensions [35]. In the United Kingdom, stress, depression, or anxiety accounted for 44% of all work-related ill health cases in 2018 [36].

Interestingly, both intervention groups used the app in a similar way. Torbjørnsen et al [37] reported similar results where the use of the app was not particularly different between the intervention groups. There is evidence from previous research that support from peers or counselors is usually an effective way to increase intervention engagement and effectiveness [38-40], but this was not observed in our study. There were some differences between the groups, however, with the reported habit performances. The habit categories with the most performances were meal frequency, positive mood, and stress management in the digital group and vegetables, stress management, and meal frequency in the F2F+digital group. This can be partly explained by the content of the face-to-face group coaching where nurses promoted the use of vegetables as part of a healthy diet.

### Implications

This study has some implications for research and practice. First, results demonstrate that internet-based lifestyle interventions can be delivered to large groups including community-dwelling middle-aged and older adults, many with limited experience in digital app use, without additional user training. Interventions can be used independently or in addition to face-to-face group coaching. Second, user engagement is critical, and possibilities to disengagement should be identified in advance and tackled with appropriate solutions such as reminders. Our results indicate that use sessions of the BitHabit app were short and relatively frequent, which was the intended way of using the app to boost habit formation by repetition of tiny behaviors. Third, the popularity of habits related to stress management and positive mood indicates that there is a huge need for solutions addressing mental health issues. Following Stein et al [41], there should be an integrated response to mental disorders and other chronic diseases in health systems because mental disorders share common features with other chronic communicable and noncommunicable diseases.

### Limitations

This study has some limitations. We cannot yet know if the weekly engagement was enough to change lifestyles of the participants because the effective engagement is not yet known and needs to be determined empirically when the outcomes are available [30]. According to Miller et al [42], engagement to digital interventions is a multidimensional concept, including both the extent to which an intervention is used and the subjective experience of the user. Unfortunately we did not have qualitative data from the first 6 months of the study available for analysis, but it would have been valuable to inform explanations for the patterns observed. One possible user experience problem is that the app offered a broad selection of BitHabits with tailoring affecting the order of categories but not the content. Although it was expected that the abundance of suggestions ensured that there was enough variety for each user and quick browsing would ensure users could easily find what was relevant for them, some users might have expected more personalization.

### Recommendations for Further Research

There are many possibilities to further research. Following the logic of habit theories that suggest complex tasks may be less prone to become automatic than simple tasks [9] and adapting the tiny habit concept by Fogg [20], the BitHabits presented by the app were designed to be simple enough and contextualized to be carried out in the participants’ daily lives. It will be important to study whether participants were able to form spans and paths as expected and how these link with habit automaticity measures [43] included in our study questionnaire. Furthermore, in order to understand habit formation better, we will need to analyze our use and questionnaire data more carefully to identify determinants for use trajectories. Our rich data will provide unique opportunities to analyze behavior change processes.

### Conclusion

Our aim was to develop a scalable solution as a tool for lifestyle modification for type 2 diabetes prevention that could be adopted easily by community-dwelling middle-aged and older adults, many with limited experiences in digital app use, without additional user training to promote users’ autonomy and help them change their habits. We found that our solution was accessible by the participants with their own smart devices and almost all tried out the basic functionality of selecting habits and reporting them. This intermediate analysis of use behavior showed relatively good engagement, with the percentage of active weekly users remaining over 50% at 6 months. However, we cannot yet know if the weekly engagement was enough to change the lifestyles of participants. Sufficient engagement with the intervention to achieve intended outcomes (ie, effective engagement) still needs to be determined empirically when outcomes are available [30]. A total of 1,089,555 habit performances were reported during the first 6 months. Categories related to the nontraditional type 2 diabetes risk factors stress management and positive mood were among the most popular ones in both groups.

## Appendix

Multimedia Appendix 1 System description.

Multimedia Appendix 2 CONSORT-eHEALTH checklist (V 1.6.1).

## Abbreviations

F2F+digital: face-to-face coaching plus digital intervention
IQR: interquartile range
RCT: randomized controlled trial
SDT: self-determination theory
