# Supplementary material for: Internet-Based Lifestyle Intervention to Prevent Type 2 Diabetes Through Healthy Habits: Design and 6-Month Usage Results of Randomized Controlled Trial
Source: JMIR Diabetes. 2020 Aug 11;5(3):e15219. doi: 10.2196/15219 (PMC7448183; doi:10.2196/15219)
Supplement: Multimedia Appendix 1 [file diabetes_v5i3e15219_app1.pdf]

Multimedia Appendix 1: System description

As a whole, the system is consisted of a Content Management System (CMS) for intervention designers, app for the study participants, email and SMS reminders as well as web-services and databases that enable the functionality of the solution (see Figure 1). The system was developed by the research organizations involved in the project, but the user interface design was subcontracted from a company. The content was “frozen” during the trial, but the pop up -functionality provided dynamic content. When the General Data Protection Regulation (GDPR) went into effect in Europe, participants were informed about the collection and use of their personal data as part of the use instructions. Quality assurance methods during the Randomized Controlled Trial (RCT) included server monitoring and email-based customer support. There were only few technical problems reported by the participants and usually they were solved by clearing the cache of the web browser.

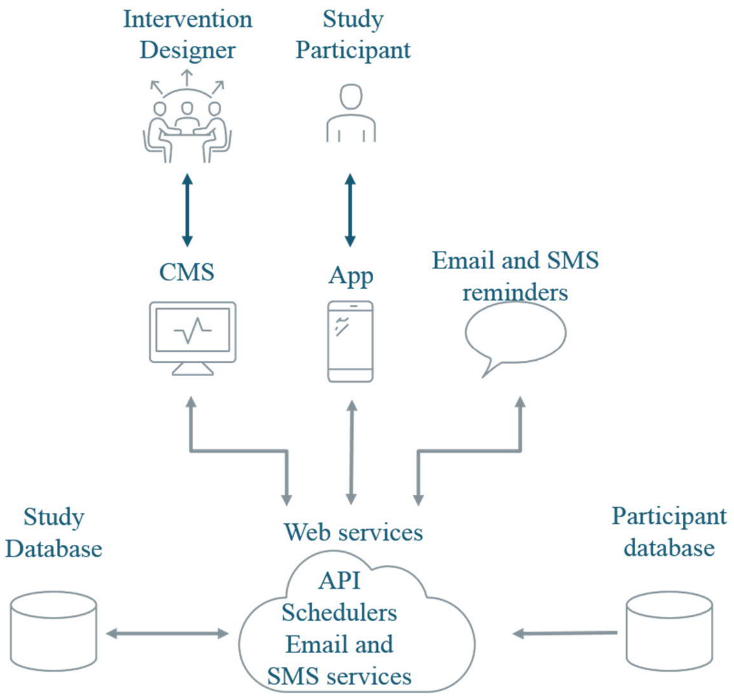

Figure 1. System description

CMS enables management of intervention content and test users including adding, modifying and deleting information. It also provides a list of study participants and a possibility to resend an invitation to the app.

Study database stores information of the app use containing major user interactions and related timestamps, such as habits selected by the users and their performances.

Both the front-end and the back-end application services are currently deployed on Microsoft Azure Cloud computing platform. The front-end of the software does not have operating environment dependencies and can be deployed on any web-server and any operating system. The back-end of the software requires Java runtime environment. Web services read information from the participant database, automatically generate links to the app for new users, take care of all SMS, email and pop up -functionality, monitor the app use and communicate with the study database.

Participant database holds the list of randomized study participants. The web services read limited set of user information from the participant database without permission to modify the information.
